# Psychological distress, neuroticism and disability associated with secondary chronic headache in the general population – the Akershus study of chronic headache

**DOI:** 10.1186/s10194-018-0894-7

**Published:** 2018-08-03

**Authors:** Espen Saxhaug Kristoffersen, Kjersti Aaseth, Ragnhild Berling Grande, Christofer Lundqvist, Michael Bjørn Russell

**Affiliations:** 1 Department of General Practice, Institute of Health and Society, University of Oslo, Box 1130 Blindern, 0318 Oslo, PO Norway; 2 Head and Neck Research Group, Research Centre, Akershus University Hospital, Lørenskog, Norway; 3 The National Center for Epilepsy, Oslo University Hospital, Oslo, Norway; 4 Institute of Clinical Medicine, Campus Akershus University Hospital, University of Oslo, Nordbyhagen, Norway; 5 HØKH, Research Centre, Akershus University Hospital, Lørenskog, Norway; 6 Department of Neurology, Akershus University Hospital, Lørenskog, Norway

## Abstract

**Background:**

Primary headaches are associated with psychological distress, neuroticism and disability. However, little is known about headache-related disability and psychological distress among people with secondary chronic headaches.

**Methods:**

30,000 persons aged 30–44 from the general population was screened for headache by a questionnaire. The responder rate was 71%. The International Classification of Headache Disorders with supplementary definitions for chronic rhinosinusitis and cervicogenic headache were used. The Hopkins Symptom Checklist-25 assessed high psychological distress, the Migraine Disability Assessment questionnaire assessed disability, and Eysenck Personality Questionnaire assessed neuroticism.

**Results:**

Ninety-five of the 113 eligible participants (84%) completed the self-reported questionnaire. A total of 38 people had chronic post-traumatic headache, 21 had cervicogenic headache, and 39 had headache attributed to chronic rhinosinusitis, while 9 had co-occurrence of chronic post-traumatic and cervicogenic headache. Six persons had miscellaneous secondary chronic headaches. Overall, 49% of those with secondary chronic headache reported high psychological distress, which is significantly higher than in the general population. A high level of neuroticism was significantly more common in those with secondary chronic headache than in the general population. Severe headache-related disability was reported by 69%. 92 persons were followed up after 3 years. A low headache frequency was the only significant predictor of improvement of ≥ 25% in headache days. Having post-traumatic or cervicogenic headache and not headache attributed to chronic rhinosinusitis predicted an increased risk &gt; 25% worsening of headache days or having a severe disability at 3 years follow-up.

**Conclusion:**

Psychological distress and neuroticism were more common among people with secondary chronic headache than in the general population. Only a high headache frequency was significantly associated with increased headache disability at baseline and a poor prognosis in the long term.

## Background

Headache, anxiety and depression are all prevalent conditions in the general population [1]. It has been suggested that anxiety, depression and neuroticism scores are associated with primary headaches [2–6]. Whether or not these factors are associated as a cause or a consequence of the headaches is still debated and personality traits and psychological problems may influence cognitive and affective functioning [3, 7, 8]. Neuroticism is often described as a personality trait that reflects the extent to which a person experiences the world as stressful, anxious, threatening, and problematic [9, 10]. Furthermore, neuroticism has been associated with depression [11].

Secondary headache after head traumas, whiplash, neck conditions or rhinosinusitis resolves in the majority of cases. The reasons why some people develop more persistent symptoms are disputed [12–16]. In addition, the pathophysiology of most of these secondary headache disorders is poorly understood [15, 16]. Both the existence of psychological distress and neuroticism are associated with chronic pain conditions and have been suggested to play important roles in the transition from acute to chronic pain in cognitive and behavioural models [9, 10, 17, 18]. Furthermore, anxiety, depression, psychological distress and neuroticism are considered to be vulnerability factors that lower the threshold at which pain is perceived as threatening, thus contributing to pain-catastrophizing and anxiety, which are associated with the progression of chronic pain [9, 10, 17, 18]. In addition, affective temperaments, personality traits, perceptions and psychological distress may significantly and negatively modify disability, treatment outcomes and long-term prognosis of patients with headache and chronic pain conditions [19–23].

However, neither anxiety, depression, neuroticism nor headache-related disability have been studied in secondary chronic headache.

The main aim of the present study was to investigate psychological distress, neuroticism and disability in people with secondary chronic headache from the general population. A secondary aim was to evaluate whether psychological distress, neuroticism or disability predicted the long-term prognosis of secondary chronic headache.

## Methods

### Study design, population and variables

This was a cross-sectional epidemiological survey of 30,000 representative persons aged 30–44 drawn from the general population of eastern Akershus County, Norway [24]. A postal questionnaire screened for possible chronic headache (≥15 days/last month and/or ≥ 180 days/last year). Screening-positive subjects were invited to a clinical interview at Akershus University Hospital.

The sample size was reduced to 28,871 because of error in the address list (*n* = 1065), emigration (*n* = 32), multi-handicap (*n* = 28), insufficient Norwegian language skills (n = 2) and death (n = 2). In total 71% (20,598/28,871) of the study population responded to the screening questionnaire. Among responders, the first questionnaire, and second and third reminders were replied to by 64%, 23% and 13%, respectively. There was no significant difference between self-reported chronic headache and response to the three reminder waves when analysed separately by sex.

Of 935 people with self-reported chronic headache, 53 persons did not consent to further contact, and 30 persons did not speak Norwegian. Among the 852 eligible, 139 declined participation and 80 could not be reached by telephone. In total, 633 participated in clinical interviews (490 as an ambulatory visit, 143 by telephone).

**Fig. 1:**
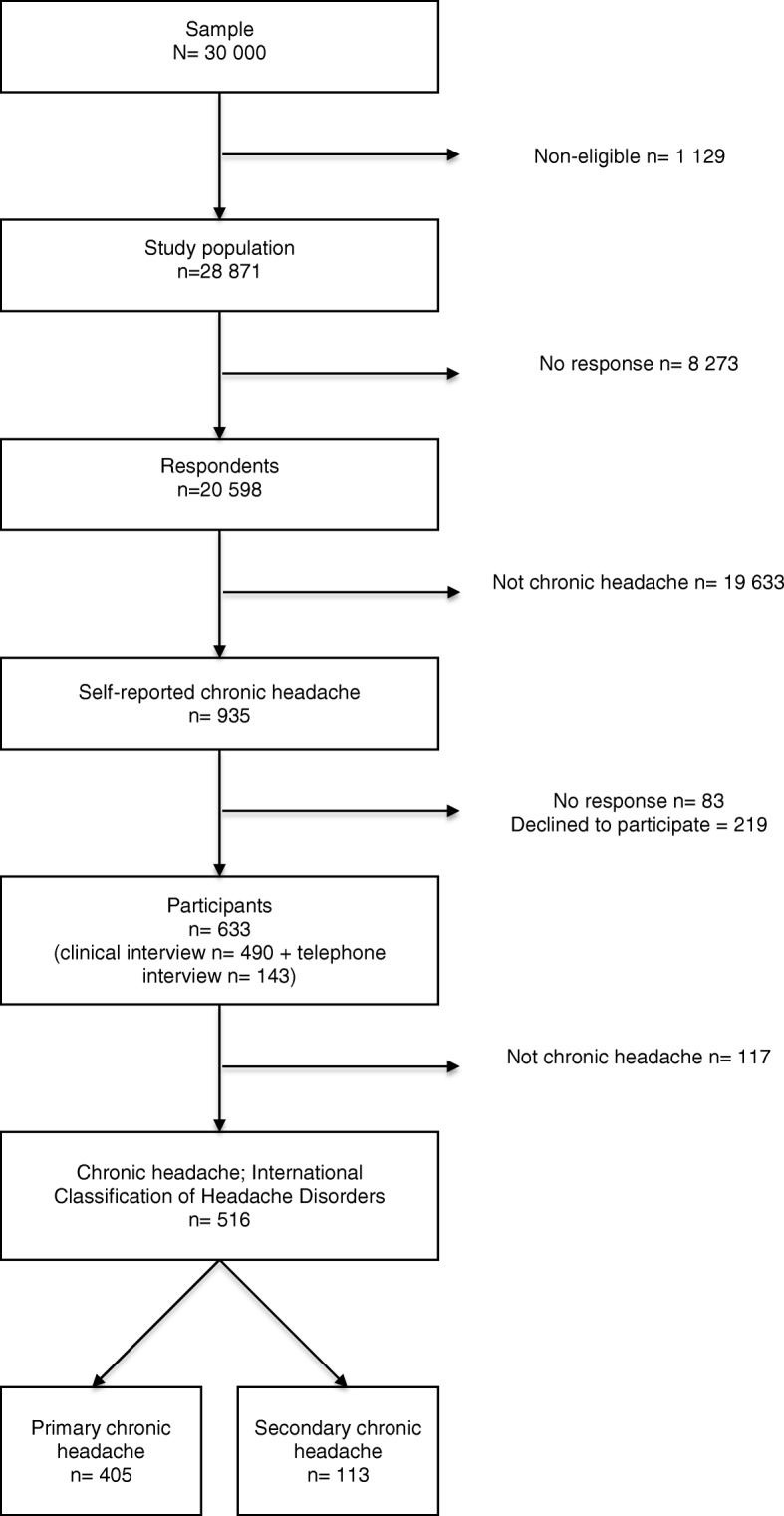
Flowchart of the study

Figure 1 shows a flow chart of the study.The method has been described in detail elsewhere [24, 25].

After the interview, the participants filled in a self-administered questionnaire including the Hopkins Symptom Checklist-25 (HSCL-25), The Migraine Disability Assessment (MIDAS) questionnaire and the Eysenck Personality Questionnaire (EPQ) N- and L-scale. The participants also provided information on socio-demographics, height, weight, smoking status, medication-overuse and headache frequency.

Semi-structured follow-up interviews were conducted after an average of 3 years, mainly by telephone (by RBG and KAA) [26]. Among the 113 persons who fulfilled the inclusion criteria, 9 persons were not eligible because of unavailable telephone numbers, and 12 did not answer the telephone despite at least six attempts. Thus, 92 people were followed-up after 3 years.

### Inclusion criteria

Secondary chronic headache.

### Exclusion criteria

Secondary chronic headache exclusively due to medication overuse.

### Headache classification

The International Classification of Headache Disorders (ICHD-II) was applied based on the interviews, and the diagnoses were later reclassified according to ICHD-III [27].

Chronic headache was defined as headache ≥15 days/months for at least 3 months or ≥ 180 days/year. Chronic post-traumatic headache (CPTH) included head (*n* = 24) and whiplash (*n* = 14) traumas. Among those with CPTH caused by head injury, 20 had a mild head injury and four had a moderate to severe head injury. Cervicogenic headache (CEH) was additionally classified according to the criteria of the Cervicogenic Headache International Study Group, requiring at least three criteria to be fulfilled, not including blockade of the neck due to the non-interventional nature of our study (Textbox 1a) [28]. Headache attributed to chronic rhinosinusitis (HACRS) was also, in addition, defined according to the criteria established by the American Academy of Otolaryngology – Head and Neck Surgery (Textbox 2b) adding that the symptoms had persisted for 12 weeks or more [29].

### Anxiety, depression and psychological distress

The Hopkins Symptom Checklist-25 (HSCL-25) explores the symptoms of depression and anxiety and is a validated tool for measuring the level of psychological distress [30]. The HSCL-25 corresponds well to DSM-IV defined depression and anxiety disorders, depression, phobia and somatoform illness using “the Composite International Diagnostic Interview” (CIDI) as gold standard diagnostic instrument [30–32]. The 25 items are scored on a scale from 1 (not bothered) to four (extremely bothered). If 20 or more of the 25 items were answered, a mean score was calculated. High psychological distress was defined as a mean HSCL-25 score ≥ 1.67 for men and ≥ 1.75 for women [31]. Although the HSCL-25 measures anxiety and depression dimensions, “forced” two-factor analyses are in favour of a one-factor solution [31]. Thus, in the present study both the anxiety, depression and the mean total HSCL-25 scores are given, but we only used the mean total HSCL-25 score to define psychological distress which, thus includes both anxiety disorder and depression.

### Neuroticism

The Eysenck Personality Questionnaire (EPQ) is an instrument designed to measure personality dimensions or traits [33]. We used two of the four EPQ scales: the 23-item neuroticism scale (N-scale) and the 21-item lie scale (L-scale) to assess neuroticism.

The EPQ N-scale is designed to measure neurotic personality traits and symptoms of moodiness, nervousness, being easily irritated, lack of endurance, and feelings of guilt and worry [33]. The L- scale assesses dissimulation or a tendency toward social conformity [33]. Both scales are dichotomous, yes or no. ‘Yes’ was scored as 1 and ‘no’ was scored as 0.

A low N-scale score indicates a low level of neuroticism and a high L-scale score indicates a high level of social conformity. EPQ has previously been validated in Norway [34].

### Headache-related disability

The Migraine Disability Assessment (MIDAS) questionnaire is a valid and widely used instrument to measure headache disability [35].

MIDAS consists of five questions concerning headache and the number of days, in the past 3 months, of activity limitations (impairment in role functioning) in three domains: schoolwork or work for pay; housework; and family, social, or leisure activities. Disability grade was scored according to MIDAS as minimal (0–5), mild (6–10), moderate (11–20) or severe (≥ 21) [35].

### HSCL-25 and EPQ population controls

The age- and sex-matched HSCL-25 scores reported from the general population in this study were derived from the cross-sectional Oslo-Lofoten 2001 study [6, 36]. This study was designed to examine general health and mental health within two geographically diverse areas, one urban (Oslo) and one rural (Lofoten). The participants were interviewed with a fully structured interview that assessed a broad range of topics related to mental and physical health [6, 36].

The EPQ scores were derived from a cross-sectional Danish study of headache disorders in the general population [5]. This sample was representative of the Danish general population.

### Statistical analysis

For descriptive data, proportions, means and standard deviations (SD) or 95% confidence intervals (CI) are given. Groups were compared using the *t*-test (continuous data) or the *χ*^2^ test (categorical data).

Logistic regression models were used to evaluate presence of i) high psychological distress and ii) severe disability at baseline in secondary chronic headaches. Linear regression was used to investigate the association between neuroticism and secondary chronic headache. High psychological distress and neuroticism were clearly correlated with a high degree of collinearity and therefore not used in the same regression analysis. Furthermore, logistic regression was also used to evaluate i) reversion to episodic headache and ii) headache improvement (≥ 25% reduction in headache days), iii) headache worsening (≥ 25% increase in headache days), and iv) severe headache disability (MIDAS≥ 21) after 3 years follow-up. The results are presented with odds ratios (ORs) with 95% CIs.

As this was a hypothesis generating descriptive study Bonferroni corrections were not done and significance levels were set at *p* &lt; 0.05, using two-sided test. All statistical analyses were performed using SPSS version 25.0.

### Ethical issues

The Regional Committee for Medical Research Ethics and the Norwegian Social Science Data Services approved the study. All participants gave informed consent.

## Results

In total 95 of the 113 eligible participants (84%) completed the self-reported questionnaire at baseline. Responders and non-responders did not differ in age, gender, or in the distribution of headache diagnoses (data not shown).

A total of 38 people had CPTH, 21 had CEH and 39 had HACRS, while 9 had co-occurrence of CPTH and CEH. Six persons had miscellaneous secondary chronic headaches. Those with CPTH and CEH were descriptive similar (gender, co-occurrence of migraine, medication overuse) and were also due to small groups merged for the purpose of statistical analyses. Descriptive characteristics of the sample are given in Table 1.

**Table 1:** Descriptive statistics for all respondents with secondary chronic headache. *P*-value given for the comparison of chronic post-traumatic headache/cervicogenic headache vs. headache attributed to chronic rhinosinusitis

	All secondary chronic headaches *N* = 95	Post-traumatic/cervicogenic headache *N* = 50	Rhinosinusitis headache *N* = 39	*(p-value for CPTH/CEH* vs. *HACRS)*
Age, mean (SD)	38.7 (4.2)	38.9 (4.2)	38.9 (3.8)	0.82
Gender, n (%)				0.06
Female	77 (81)	37 (74)	35 (90)	
Male	18 (19)	13 (26)	4 (10)	
Education, highest attained, n (%)				0.92
≤ 15 years	75 (76)	38 (76)	30 (77)	
&gt; 15 years	24 (25)	12 (24)	9 (23)	
Married or cohabitant, n (%)	60 (63)	32 (64)	25 (64)	0.99
Body mass index (kg/m^2^), mean (SD)	26.6 (5.0)	27.4 (5.3)	25.5 (4.5)	0.11
Daily smoker, n (%)				0.93
No	57 (63)	30 (63)	24 (62)	
Yes	35 (37)	18 (37)	15 (38)	
Concomitant migraine, n (%)				0.15
No	55 (58)	32 (64)	19 (49)	
Yes	40 (42)	18 (36)	20 (51)	
Number of headache days past 3 months, mean (SD)	62.0 (27.1)	70.5 (25.4)	52.4 (24.5)	0.002
Number of medication days past month, mean (SD)	12.8 (10.8)	13.4 (11.5)	13.2 (10.3)	0.94
Medication-overuse, n (%)				0.76
No	50 (53)	26 (52)	19 (49)	
Yes	45 (47)	24 (48)	20 (51)	
HSCL-25 scores, mean (SD)				
Anxiety score, mean (SD)				
Female	1.80 (0.46)	1.88 (0.43)	1.79 (0.48)	0.37
Male	1.84 (0.49)	1.85 (0.50)	1.88 (0.56)	0.94
Depression score, mean (SD)				
Female	1.83 (0.55)	1.88 (0.59)	1.87 (0.50)	0.96
Male	1.79 (0.56)	1.76 (0.56)	1.67 (0.49)	0.77
Total score, mean (SD)				
Female	1.82 (0.48)	1.88 (0.49)	1.84 (0.44)	0.70
Male	1.81 (0.49)	1.80 (0.52)	1.75 (0.51)	0.88
HSCL-25, psychological distress, n (%)				0.40
No (&lt; 1.67 for men and &lt; 1.75 for women)	48 (51)	22 (45)	21 (54)	
Yes (&gt; 1.67 for men and &gt; 1.75 for women)	46 (49)	27 (55)	18 (46)	
EPQ N-score, mean (SD)	11.4 (5.4)	10.6 (5.5)	12.9 (4.9)	0.06
EPQ L-score, mean (SD)	10.6 (3.2)	10.4 (3.6)	10.9 (2.6)	0.5
MIDAS score, mean (SD)	66 (60)	80 (60)	44 (46)	0.005
MIDAS score (grade), n (%)				0.23
0–5 Minimal	16 (20)	6 (14)	9 (27)	
6–10 Mild	4 (5)	2 (5)	2 (6)	
11–20 Moderate	5 (6)	1 (2)	3 (9)	
&gt; 20 Severe	55 (69)	33 (79)	19 (58)	

### Psychological distress and neuroticism

The anxiety and depression HSCL-25 scores were high in secondary chronic headaches (Table 1). Mean total HSCL-25 scores for women and men were 1.82 (95% CI 1.71–1.93) and 1.81 (1.56–2.05) and thus statistically significantly higher than in the general population (women; 1.39 (1.34–1.43), men; 1.25 (1.22–1.29). In total, 46% (35–57) women and 61% (39–80) men of the sample had high psychological distress, which is statistically significantly higher than 14% (10–18) of women and 9% (6–13) of men in the general population.

Neither age, gender, headache frequency, co-occurrence of migraine, medication overuse or secondary headache diagnosis (CPTH/CEH versus HACRS) were significantly associated with high psychological distress in the multivariate regression analyses (Table 2).

**Table 2:** Odds for having high psychological distress defined as mean HSCL-25 score ≥ 1.67 for men and ≥ 1.75 for women. Logistic regression

Covariate	High psychological distress							
	Bivariate (*n* = 80–94)				Multivariable (*n* = 83)			
	n	Odds ratio	95% CI	p-value	n	Odds ratio	95% CI	*p*-value
Age	94	1.0	0.9–1.1	0.5	83	1.0	0.9–1.2	0.6
Gender								
Male	18	1			17	1		
Female	76	0.5	0.2–1.6	0.3	66	0.7	0.2–2.2	0.5
Headache days last 3 months								
&lt; 80 days	50	1			47	1		
≥ 80 days	38	1.3	0.6–3.0	0.5	36	1.1	0.4–3.1	0.9
Co-occurrence of migraine								
No	54	1			48	1		
Yes	40	0.6	0.3–1.5	0.3	35	0.6	0.2–1.4	0.2
Type of headache								
HACRS	39	1			36	1		
CPTH/CEH	49	1.4	0.6–3.3	0.4	47	1.1	0.4–3.0	0.8
Medication overuse								
No	49	1			43	1		
Yes	45	2.0	0.9–4.5	0.1	40	1.9	0.8–4.8	0.2

Neuroticism as assessed by EPQ N-scale was significant higher in secondary chronic headache than in the general population (11.4 vs. 6.2, *p* &lt; 0.0001). Neither age, gender, migraine, medication overuse, type of secondary chronic headache, headache frequency nor disability were significantly associated with a high level of neuroticism in bivariate and the multivariate linear regression analyses (Table 3). The EPQ L-scale was not significantly different between those with secondary chronic headache and the general population. High psychological distress and neuroticism were not associated with co-occurrence of other chronic pain conditions.

**Table 3:** Linear regression analysis with variables associated with neuroticism in secondary chronic headache

	Neuroticism Eysenck N-scale							
	Bivariate (*n* = 79)				Multivariable (*n* = 72)			
	N	Unstandarized coefficient	95% CI	*p*-value	N	Unstandarized coefficient	95% CI	*p*-value
Age	79	0.14	−0.15; 0.43	0.3	72	0.10	−0.23; 0.42	0.6
Gender								
Male*	16	0			15	0		
Female	63	1.34	−1.62; 4.30	0.4	57	0.88	−2.53; 4.30	0.6
Headache days last 3 months								
&lt; 80 days*	41	0			40	0		
≥ 80 days	33	−0.77	−3.29; 1.76	0.5	32	−0.34	−3.43; 2.76	0.8
Co-occurrence of migraine								
No*	49	0			43	0		
Yes	30	0.09	−2.39; 2.55	0.9	29	−0.34	−3.10; 2.43	0.8
Type of headache								
HACRS*	32	0			30	0		
CPTH/CEH	44	−2.23	−4.65; 0.19	0.07	42	−2.10	−5.27; 1.07	0.2
Medication overuse								
No*	42	0			37	0		
Yes	37	1.73	−0.64; 4.09	0.2	35	1.72	−0.96; 4.39	0.2

*denotes reference group

### Headache disability

The mean MIDAS score was 66 (52–79) for secondary chronic headaches with a significantly higher mean score in CPTH/CEH than in HACRS (80 vs. 44, *p* = 0.005, Table 1). Almost 70% of those with secondary chronic headache were classified in the most severe disability class, i.e. approximately 80% of those with CPTH/CEH, and 60% of those with HACRS. This was statistically significant (*p* = 0.05). Only a high baseline headache frequency was associated with severe disability with an OR 4.3 (1.2–15.5, *p* = 0.021) in the multivariate regression analyses (Table 4).

**Table 4:** Odds for having severe disability defined as MIDAS score &gt; 20. Logistic regression

Covariate	Severe disability							
	Bivariate (*n* = 74–94)				Multivariable (n = 74)			
	n	Odds ratio	95% CI	*p*-value	n	Odds ratio	95% CI	*p*-value
Age	80	1.0	0.9–1.1	0.7	74	1.0	0.9–1.2	1.0
Gender								
Male	18	1			17	1		
Female	62	1.1	0.4–3.5	0.8	57	1.5	0.4–5.9	0.5
Headache days last 3 months								
&lt; 80 days	45	1			42	1		
≥ 80 days	34	4.2	1.4–13.0	0.01	32	4.0	1.1–14.5	0.04
Co-occurrence of migraine								
No	50	1			46	1		
Yes	30	1.1	0.4–2.9	0.9	28	1.1	0.3–3.3	0.9
Type of headache								
HACRS	33	1			32	1		
CPTH/CEH	42	2.7	1.0–7.4	0.05	42	1.9	0.6–6.4	0.3
Medication overuse								
No	44	1			39	1		
Yes	36	1.3	0.5–3.5	0.5	35	1.3	0.4–4.0	0.7
HSCL-25 defined psychological distress								
No	43	1			38	1		
Yes	37	1.5	0.6–3.8	0.5	36	1.1	0.4–3.4	0.9

### Psychological distress, neuroticism and disability as predictors of headache prognosis

In total, 78 of the 92 eligible participants (85%) at 3 years follow-up had completed the self-reported questionnaire at baseline and were available for the predictor analyses over time.

Low headache frequency (below 75th percentile, i.e. below 80 headache days over 3 months) and non-severe disability at baseline significantly predicted reversal from chronic to episodic headache in the multivariate regression analyses (Table 5). A low headache frequency at baseline was the only predictor associated with an improvement of ≥ 25% in headache days over the follow-up time period in the multivariate regression analyses (Table 5). Having CPTH/CEH and not HACRS predicted an increased risk of ≥ 25% worsening of headache days or having a severe disability at 3 years follow-up (Table 5).

**Table 5:** Multivariate logistic regression. Baseline predictors for different outcomes of secondary chronic headaches after 3 years follow-up

Covariate	No chronic headache				&gt; 25% reduction in headache days				&gt; 25% increase in headache days				Severe disability			
	Multivariate (*n* = 63)				Multivariate (*n* = 61)				Multivariate (n = 61)				Multivariate (*n* = 46)			
	N	Odds Ratio	95% CI	*p*-value	N	Odds Ratio	95% CI	*p*-value	N	Odds Ratio	95% CI	*p*-value	N	Odds Ratio	95% CI	*p*-value
Age	63	1.0	0.8–1.2	0.8	61	1.0	0.8–1.2	0.9	61	1.0	0.8–1.2	0.8	46	1.3	1.0–1.8	0.1
Gender																
Male	13	1.7	0.3–11.4	0.6	12	1.3	0.2–8.9	0.8	12	0.3	0.0–2.6	0.3	10	0.2	0.0–10.8	0.5
Female	59	1			49	1			49	1			36	1		
Headache days last 3 months																
&lt; 80 days	35	11.0	1.2–99.7	0.03	34	14.3	1.6–127.7	0.017	34	0.0	0.0–0.0	1.0	31	0.4	0.1–4.2	0.5
≥ 80 days	28	1			27	1			27	1			15	1		
Type of headache																
HACRS	28	2.6	0.5–13.3	0.3	27	2.5	0.5–11.9	0.3	27	0.1	0.0–0.8	0.03	26	0.1	0.0–0.8	0.035
CPTH/CEH	35	1			34	1			34	1			20	1		
Medication overuse																
No	32	1.6	0.3–7.3	0.6	30	1.6	0.4–7.0	0.5	30	0.2	0.0–1.6	0.2	24	0.1	0.0–1.6	0.1
Yes	31	1			31	1			31	1			22	1		
HSCL-25 defined psychological distress																
No	34	1.8	0.4–8.5	0.4	33	1.5	0.3–6.2	0.6	33	0.6	0.1–2.9	0.5	25	0.7	0.1–6.5	0.8
Yes	29	1			28	1			28	1			21	1		
Disability grade																
No to moderate	21	3.6	0.9–14.3	0.04	20	1.3	0.3–5.1	0.7	20	1.4	0.3–6.8	0.6	18	0.0	0.0–0.0	1.0
Severe	42	1			41	1			41	1			28	1		

## Discussion

In this large population-based study almost half of the subjects with secondary chronic headache reported high psychological distress. The main finding was that the prevalence of high psychological distress and neuroticism was higher than in the general population and that there were no differences between those with CPTH/CEH and HACRS regarding psychological distress and neuroticism. In terms of long term prognosis, we found that low headache disability, low headache frequency and having HACRS at baseline, but not psychological distress and neuroticism, predicted headache improvement.

### Methodological discussion

The population-based sample in the present study was large, and the high response rate ensures representativity compare to the general population aged 30–44. The age range in our study was chosen in order to ascertain little co-morbidity of non-headache disorders and use of non-headache medications.

We used population-based reference populations for comparison to minimize selection bias. The reference populations were representative for the Danish and the Norwegian general populations regarding age, gender and marital status. The Danish reference population had a wider age range than our sample, and the data was besides collected 15 years earlier. However, the latter is probably not a source for bias, as personality traits are regarded as stable over time.

Even though the sample size of secondary chronic headache is relatively small and conferred some challenges due to reduced power in the statistical analyses, it is the largest population based sample reported so far. The sample size limited the number of variables that could be included in the multivariate analyses, and forced us to dichotomize some variables. Recall bias regarding headache days and medication days cannot be excluded, but the meticulous interview and pre-completed medication list are likely to reduce such bias.

Our study is strengthened by face-to-face interviews by headache experts as this provides more valid headache diagnoses than questionnaire-based diagnoses [37].

The majority of the participants completed a full diagnostic interview conducted by a headache expert albeit with a smaller portion by telephone. The main reason for not completing a full clinical interview was not being available for travelling to a clinical interview during an ordinary work week. However, the headache diagnoses were not significantly different in these two groups of participants. Furthermore, no significant differences between data collected at the clinic and by telephone by a trained headache expert was reported in a previous study [38].

The diagnostic criteria of CEH and HACRS have been discussed for many years. At the 1st data collection the ICHD-II criteria were available, but the criteria for CEH were vague, and HACRS was not recognized as a cause of chronic headache. Thus, to improve the diagnostic accuracy we used supplementary definitions [28, 29]. All subjects diagnosed with CEH or HACRS in the present study fulfil the new ICHD-III criteria for these chronic headaches [27]. Since two physicians conducted the investigations, inter-observer variation is a possibility. However, the headache diagnoses were equally frequent by both physicians, suggesting that inter-observer variation was low.

### Psychological distress, neuroticism and disability in secondary chronic headaches

No previous study has investigated psychological distress or neuroticism for secondary chronic headache in the general population. We have previously shown that for chronic tension-type headache (CTTH) in the general population, the overall prevalence of psychological distress was 59% (53–65) for women and 43% (32–55) for men [6]. Furthermore, the mean HSCL score was 1.71 (1.60–1.82) for men with CTTH and 1.93 (1.86–2.00) for women with CTTH. Those with CTTH and co-occurrence of migraine and/or medication overuse did not have a higher level of HSCL score or psychological distress compared with those without co-occurrence of migraine and/or medication overuse [6]. These findings indicate that there are no significant differences in psychological distress between primary and secondary chronic headaches.

The prevalence of psychological distress in the Norwegian general population using the same cut-offs and age group as in the present study were 14% (10–18) for women and 9% (6–13) for men [6]. Thus, the prevalence of psychological distress is more than four times higher in people with secondary chronic headache than in the general population. A difference in psychological distress between episodic and chronic headache has been reported suggesting that the relationship between psychological problems and headache depends more on headache frequency than the type of headache [3, 39, 40]. Neither in the present study nor in a study of CTTH did more headache days above 15 days increase the odds for more psychological distress [6]. Therefore, it may be the complex burden of chronic headache or an underlying vulnerability, more than the specific headache condition or additional headache days &gt; 15 that are associated with psychological problems.

Recent reviews have estimated the global prevalence of depression and anxiety in the range 4.4–5.0% and 4.8–10.9% which is in accordance with findings from the Norwegian general population using HSCL-25 and CIDI [1, 32]. Our results thus suggest population-derived secondary chronic headache patients to lie much higher than the general population.

Whether anxiety and depression have a shared mechanism with headache, whether they represent risk factors for headache chronification or are just comorbid symptoms related to a disabling headache situation is still a matter of debate [7, 8, 27]. It may be that an improvement in headache frequency improves depression and anxiety levels or vice versa. However, independently of the causal directions of these associations, it is important always to take psychological factors into account when treating headache, as the condition is clearly associated with such factors [3, 7, 27, 39]. Thus, a best possible treatment approach for many headache sufferers includes acute and prophylactic medications and multidisciplinary treatment addressing the psychological factors such as anxiety and depression.

In the present study we report that CPTH/CEH and HACRS had similar prevalence of psychological distress despite different pathophysiological mechanisms. Furthermore, the prevalence of psychological distress was comparable to that of two other chronic headaches; chronic tension-type headache and medication-overuse headache [6, 41]. Depression and anxiety are known to be associated also with other chronic pain conditions [42–44]. However, such co-morbidity of other chronic pain did not increase the psychological distress in the present study.

The neuroticism score in secondary chronic headache reported here was comparable to that of chronic tension-type headache and chronic headache, but higher than the score reported in episodic headache [2, 5, 6]. A higher neuroticism score in primary headaches than in the general population has previously been reported with some studies suggesting a stronger association with tension-type headache than with migraine [2, 4–6]. The EPQ L-score was not significantly different from the general population and was similar to that previously reported in episodic and chronic headache [2, 5].

Disability is an important outcome as it reflects the burden and impact of diseases on daily activities [35]. Almost 70% of our participants had severe disability, suggesting that people with secondary chronic headache are among the most disabled headache patients. Surprisingly, high psychological distress, which may add to the burden of headache and pain, was not associated with increased disability.

### Prognosis of secondary chronic headaches

We have previously shown that secondary chronic headaches have varying courses, depending on the subtype, with HACRS having a better long-term prognosis than CEH [26]. However, why some people develop persistent symptoms in the first place and what predicts poor prognosis in whiplash-associated traumas, mild to moderate head injuries and neck disorders is disputed partly due to inconsistent findings [12, 13, 16, 42, 45]. The lack of correspondence between severity of the traumas (whiplash and post-traumatic headache) and neck conditions (CEH) and the chronicity of symptoms has led to the assumption that psychological factors may play a crucial role in the cause and maintenance of these disorders. However, psychological factors account for only a portion of the variance in most of these studies, thereby highlighting the possible and complex bio-behavioural pathophysiology which may partly explain these conditions [42]. It has been hypothesised that a certain set of personality traits or distress makes these patients more vulnerable, with poorer adjustment to their medical condition than other people without these personality traits [10]. Our results indicate that only a high headache frequency, severe headache disability and type of secondary headache seem to influence the outcome after 3 years. However, based on the study design, it is not possible to say if personality traits or psychological distress are linked to the development of the secondary chronic headache.

Although medication overuse was not a prognostic factor or associated with neuroticism or high psychological distress, it is worthwhile to notion that about half of all patients overused acute headache medication. Whether detoxification may help these patients with other secondary headaches is still a matter of debate.

Neuroticism may influence pain sensitivity and pain perception, and hypothetically thus be involved in a possible central sensitisation in, and prognosis of, chronic pain conditions such as chronic headaches [5, 9, 46]. The results from our follow-up study do not suggest that neuroticism predicts prognosis of secondary chronic headaches. Furthermore, in this population, reported psychological distress also does not predict whether secondary chronic headaches improve or not. These findings may shed some light on the “hen and egg” issue; the headache rather than the personality characteristics seems to determine the prognosis of these secondary headaches.

## Conclusion

People with secondary chronic headache have a higher psychological distress and neuroticism score than people from the general population. In terms of prognostic findings, only headache frequency and disability predicted improvement of the secondary headache, while psychological factors did not. Thus, the prime focus should be headache management, i.e. proper medication and multidisciplinary treatment addressing the psychological factors such as anxiety, depression, distress and neuroticism.

***Textbox 1a****. Definition of cervicogenic headache* [28]*. It is obligatory that one or more of the phenomena Ia–Ic are present.*

**Table:** 

Major criteria	I. Symptoms and signs of neck involvement Ia. Precipitation of head pain, similar to the usually occurring one: Ia1) by neck movement and/or sustained, awkward head positioning, and/or: Ia2) by external pressure over the upper cervical or occipital region on the symptomatic side. Ib. Restriction of the range of motion (ROM) in the neck. Ic. Ipsilateral neck, shoulder or arm pain of a rather vague, non-radicular nature, or – occasionally – arm pain of a radicular nature. II. Confirmatory evidence by diagnostic anaesthetic blockades. III. Unilaterality of the head pain, without sideshift.
Head pain characteristics	IV. Moderate-severe, non-throbbing pain, usually starting in the neck. Episodes of varying duration, or: fluctuating, continuous pain.
Other characteristics of some importance	V. Only marginal effect or lack of effect of indomethacin. Only marginal effect or lack of effect of ergotamine and sumatriptan. Female sex. Not infrequent occurrence of head or indirect neck trauma by history, usually of more than only medium severity.
Other features of lesser importance	VI. Various attack-related phenomena, only occasionally present, and/or moderately expressed when present: a) nausea, b) phono- and photophobia, c) dizziness, d) ipsilateral “blurred vision”, e) difficulties swallowing, f) ipsilateral oedema, mostly in the periocular area.

***Textbox 1b.*** *Definition of rhinosinusitis by the American Academy of Otolaryngology – Head and Neck Surgery* [29]*. Two major factors or one major and two minor factors are required for the diagnosis. Of note, facial pain requires another major factor associated with it for diagnosis, as facial pain plus two minor factors is not deemed sufficient for diagnoses of rhinosinusitis.*

**Table:** 

*Major factors*	
Facial pain/pressure	
Nasal obstruction/blockage	
Nasal discharge/purulence/discolored postnasal drainage	
Hyposmia/anosmia	
Purulence in nasal cavity on examination	
Fever (acute rhinosinusitis)	
*Minor factors*	
Headache	
Fever (all nonacute)	
Halitosis	
Fatigue	
Dental pain	
Cough	
Ear pain/pressure/fullness	

## Abbreviations

CEH: Cervicogenic headache
CI: Confidence intervals
CIDI: The Composite International Diagnostic Interview
CPTH: Chronic post-traumatic headache
CTTH: Chronic tension-type headache
EPQ: Eysenck Personality Questionnaire
HACRS: Headache attributed to chronic rhinosinusitis
HSCL-25: Hopkins Symptom Checklist-25
ICHD: The International Classification of Headache Disorders
MIDAS: The Migraine Disability Assessment
ORs: Odds ratios
